# A Road Defect Detection System Using Smartphones

**DOI:** 10.3390/s24072099

**Published:** 2024-03-25

**Authors:** Gyulim Kim, Seungku Kim

**Affiliations:** Electronics Engineering, Chungbuk National University, Cheongju 28644, Republic of Korea; kyurim8535@chungbuk.ac.kr

**Keywords:** automatic data collection, CNN, road defect detection, smartphone

## Abstract

We propose a novel approach to detecting road defects by leveraging smartphones. This approach presents an automatic data collection mechanism and a deep learning model for road defect detection on smartphones. The automatic data collection mechanism provides a practical and reliable way to collect and label data for road defect detection research, significantly facilitating the execution of investigations in this research field. By leveraging the automatically collected data, we designed a CNN-based model to classify speed bumps, manholes, and potholes, which outperforms conventional models in both accuracy and processing speed. The proposed system represents a highly practical and scalable technology that can be implemented using commercial smartphones, thereby presenting substantial promise for real-world applications.

## 1. Introduction

This paper addresses research on the classification of road defects caused by various factors, such as weather and heavy vehicle traffic. Increased rainfall during the summer months can lead to road surface erosion and pothole formation, while the repeated transit of large vehicles can cause deformation and cracking of the road. Such road defects pose a serious risk to road safety and can lead to severe accidents, necessitating prompt detection and proper maintenance [1].

Effective maintenance of road infrastructure plays a crucial role in traffic safety and socio-economic stability. Prompt detection and appropriate response to road defects are essential in several key aspects. Road defects can lead to difficulties in vehicle control and increase the risk of accidents. Driving on roads with defects can cause serious accidents for both drivers and pedestrians. Additionally, cracks or unevenness on the road can damage vehicle tires or suspension systems, leading to increased repair costs and overall vehicle performance degradation [2].

The current method of reporting road defects primarily involves getting out of the vehicle to take a picture and report the issue. This can be dangerous on highways or heavily trafficked roads, and it has the disadvantage of taking a long time for repairs. In Canada, it takes less than 3 months to repair potholes during the winter. However, in the summer, it can take more than 24 months [3]. According to the Ministry of Land, Infrastructure, and Transport of Korea, the longest time taken to address a complaint was up to 99 days, with the duration being 99 days in 2020, 96 days in 2021, and 94 days in 2022, taking more than three months for three consecutive years [4]. Therefore, research is needed on ways to easily and safely detect road defects and share accurate defect locations.

Recent research in road defect detection is categorized into three main technologies: The first is a vision-based approach using cameras, which detects road defects through image processing and deep learning. The image processing techniques are as follows: Akagic et al. [5] introduce an efficient vision-based method for pothole detection using red, green, blue (RGB) color space image segmentation, highlighting the potential of color analysis in identifying road defects. Pan et al. [6] present a solution to detect potholes using disparity transformation and road surface modeling. For detecting multiple potholes, Jeong et al. [7] propose a real-time image processing method based on watershed techniques, emphasizing the efficiency of real-time applications. Additionally, a pothole classification model that uses edge detection techniques, one of the image processing methods, for road defect classification is proposed [8]. There is research detecting potholes with thermal cameras, focusing on using deep neural networks to detect and locate depressions in thermal images, exploring further the application of advanced neural networks in thermal imaging [9]. Masihullah S. [10] presents an attention-based joint framework for road and pothole segmentation, showcasing the application of attention mechanisms in road image analysis. It also comprehensively highlights the use of Convolutional Neural Network (CNN) for pothole detection and analysis, showing various innovative approaches to road surface monitoring for safety enhancement [11,12,13,14]. These studies demonstrate the significant potential of CNN for enhancing road safety and maintenance through advanced image processing and analysis techniques. Each research project provides unique insights into the application of CNN, from thermal imaging to regional location-aware approaches, improving the detection and analysis of road potholes.

The second technique is a 3D reconstruction method that generates a three-dimensional image of the road surface, allowing for precise identification of defects such as type, size, and depth. Haq et al. [15] introduced a stereo-based 3D reconstruction method using a hybrid dense matching approach to highlight the use of stereo vision to represent the depressed areas of the road in detailed 3D. Like stereo vision, they combine deep learning to propose an automated pixel-level pavement distress detection method, emphasizing the integration of advanced imaging technology and AI for precise pavement analysis [16]. Du et al. [17] propose a pothole detection method based on 3D point cloud segmentation, explaining the use of three-dimensional spatial data for accurate pothole identification. Additionally, they suggest advanced methods for crack detection on paved roads using 3D data [18,19].

Finally, in road surface monitoring and maintenance, the role of smartphones, sensor technology, and computational methods has become increasingly important, offering cost-effective, scalable, and innovative solutions for detecting depressions and various kinds of road defects. Kyriakou et al. [20] propose to use smartphone sensors and onboard diagnostics (OBD) to detect signs of road pavement abnormalities. This approach combines Artificial Neural Network (ANN) technology with smartphone sensors to capture the interaction between moving vehicles and the road surface, using observed interaction patterns to detect depressions. This method employs four metrics for analysis and demonstrates an approximately 90% detection accuracy. Initial results, which include additional road defects in the analysis and distinguish potholes from other pavement defects, are promising. This study underscores the value of low-cost pothole detection using smartphone sensors. Furthermore, Nguyen et al.’s research [21], another smartphone-based pothole approach, extends smartphone usage beyond road monitoring to general environmental sensing. It explores how smartphones can detect environmental anomalies and demonstrates the versatile application of mobile sensors in various aspects of environmental monitoring. In a study proposed by Chao Wu et al. [22], which incorporates the Global Positioning System (GPS) into smartphones, an automated pothole detection system is introduced. They collected road condition data in urban areas using vehicles and smartphones and tested various machine learning classifiers based on this data. The Random Forest method proved to be the most effective for pothole classification, achieving a precision of 88.5% and a recall of 75%. Additionally, the system’s diversity and robustness were validated through datasets from various road types. Research is also underway to detect not only potholes but also manhole covers on roads. Zhou et al. [23] introduce a smartphone-based method for detecting and classifying road manhole covers, expanding the scope of pavement monitoring using smartphone sensors.

All road defect detection technologies have their own advantages and limitations, as summarized in Table 1. The vision-based approaches use cameras to extract images, detecting road defects through image processing and deep learning. This approach is useful for identifying the types and quantities of road defects but is heavily affected by lighting, limiting its use during nighttime or rainy weather. The 3D reconstruction methods have the advantage of providing detailed insights into the types, sizes, and depths of road defects. However, this requires the use of detecting equipment, which incurs additional costs for road defects. The vibration sensor-based road defect detection techniques are less affected by environmental influences such as weather and time and are a cost-effective solution. However, these techniques have the disadvantage of requiring a lot of preliminary data collection to ensure high accuracy.

This paper proposes a road defect detection system based on vibration sensors, specifically accelerometers. The cornerstone of our methodology is an automated system for data collection specifically designed to facilitate the classification of various road conditions essential for identifying road defects. This system is adept at distinguishing between common road defects, such as potholes, and exceptional conditions, including speed bumps and manholes, thereby generating a rich dataset for analysis. For the purposes of clarity and simplicity within this paper, all aforementioned exceptional road conditions, including but not limited to speed bumps, manholes, and potholes, will collectively be categorized and referred to as “road defects”. Leveraging accelerometer data sourced from smartphones and video footage from dashcams, our system enables the efficient collection of high-quality, labeled datasets. These datasets are crucial for the training of deep learning models aimed at road defect detection. The ease of data collection and the quality of the resulting dataset underscore the system’s utility in generating valuable inputs for deep learning algorithms. The implications of our collected data and the automated collection system are significant for the advancement of research in road defect detection using vibration sensors. Additionally, we present a deep learning model that, utilizing the automatically collected data, accurately classifies three specific road defects: speed bumps, manholes, and potholes. The remainder of this paper is organized as follows: In Section 2, we introduce conventional vibration sensor-based road defect detection technologies and derive the problems in collecting data. Section 3 explains the proposed road defect detection system, dividing it into two parts: an automatic data collection part and a road defect classification part. Section 4 covers the collection of training data using smartphones and vehicles and evaluates accuracy through a test dataset. Finally, Section 5 discusses the conclusions.

## 2. Related Work

We study a vibration sensor-based road defect detection method that is cost-effective and has a low environmental impact. Before introducing the proposed method, this section introduces conventional work on vibration sensor-based road defect detection methods and derives related problems.

### 2.1. Vibration Sensor-Based Threshold Method

The threshold-based approach utilizes inertial sensor data to detect and classify road anomalies. This method involves detecting road anomalies when certain threshold values are exceeded for signal parameters obtained from the sensors, such as amplitude, root mean square, and wavelet coefficients [24]. It presents new algorithms leveraging the hardware and software capabilities of Android devices, with a particular focus on detecting potholes in real-time events and using limited resources. The algorithms include Z-THRESH, Z-DIFF, STDEV(Z), and G-ZERO [25]. It introduces a system that automatically detects potholes and speed bumps using the Android operating system. This application of standard smartphones in identifying road anomalies emphasizes easier and more widespread access to road condition monitoring, addressing device reliability in critical applications such as road safety [26,27]. Furthermore, it goes beyond previous research by incorporating crowdsourcing methods. It discusses enhancing road anomaly detection through crowd detection, which collects data from many users. This approach demonstrates how leveraging the collective detection capability of the community can improve road condition monitoring [28,29,30].

### 2.2. Vibration Sensor-Based Machine Learning Method

Bustamante-Bello et al. [31] introduce a novel approach for visualizing road pavement anomalies using fog computing in a vehicle-to-infrastructure (V2I) network and machine learning. In this research, they propose a method to efficiently process real-time road condition data and visualize surface anomalies promptly using fog computing, enhancing the ability to detect and respond to road anomalies. The focus here lies in detecting speed bumps using features derived from accelerometer data and optimizing this process with a genetic algorithm. Additionally, they propose a method for detecting road anomalies by comparing data windows of varying lengths using Dynamic Time Warping (DTW). Expanding on the DTW method, they introduce a system named Quick Filter Based Dynamic Time Warping (QFB-DTW) that utilizes a series of accelerometer data points to discover anomalies on the road surface [32]. Wu et al. [22] propose an automated machine learning system using Random Forest to detect potholes on the road using smartphone sensor data. The significance of this research lies in the use of widely accessible smartphones and their built-in sensors to collect data that machine learning algorithms can process for road anomaly detection. Ferjani et al. [24] explore optimized machine learning techniques for road monitoring. They perform sensitivity analyses of three machine learning models, including Support Vector Machines (SVM), Decision Trees (DT), and Multilayer Perceptrons (MLPs), to test the effectiveness of feature selection. This contributes to an ongoing conversation about selecting and effectively applying algorithms for efficient and reliable road monitoring systems, which is crucial for assessing road conditions. They also describe a Smart Pothole Detection System that utilizes a One-class Support Vector Machine (OCSVM) instead of an SVM. This research explains the integration of sensor technology for vehicle-based road condition monitoring and advanced computational methods, showcasing potential directions for intelligent transportation systems [33]. Julio-Rodríguez [34] develops a classification method to enhance context awareness in autonomous vehicles. They classify driving environments using IMU sensors and energy consumption data without relying on computer vision. They evaluated 13 classification algorithms to select the optimal method. These results demonstrate the applicability of autonomous driving technology advancements in path planning and safety.

### 2.3. Vibration Sensor-Based Deep Learning Method

To accurately detect abnormalities on road surfaces, a new approach is proposed, incorporating various deep learning models, including CNN, Long Short-Term Memory (LSTM) networks, and reservoir computing models. This method utilizes crowdsourced data to distinguish potholes from other road instabilities, achieving high accuracy in real experiments [35]. Three deep learning models—the Deep Feedforward Network (DFN), CNN, and Recurrent Neural Network (RNN)—are applied. These models are trained and evaluated using data from various road anomalies collected from vehicles. Additionally, three sets of numerical features are proposed to represent road conditions, and a comparative study of the performance of each deep learning model is conducted [36]. In contrast, there is a paper that compares machine learning and deep learning approaches for detecting anomalies on road surfaces. It focuses on classifying three major road conditions: smooth roads, potholes, and deep transverse cracks. The hypothesis that using features extracted from all sensor axes provides superior classification results compared to using a single axis is tested. The performance of machine learning models, including deep neural networks, is also evaluated, confirming the effective classification of road conditions without manual feature extraction [37]. They demonstrate road surface anomaly detection and classification using crowdsourced smartphone sensor data. This research approaches road condition classification through multi-layered activities that classify road types and anomalies using a Spiking Neural Network (SNN) learning model [38].

In this paper, the goal is to design an automatic collection system for road defect training data that is more easily collected to solve the difficulty of collecting training data, which is a problem with deep learning methods.

## 3. A Proposed Road Defect Detection System

In this manuscript, we detail two significant contributions stemming from our innovative road defect detection system. Firstly, we introduce a method for the automation of data collection and labeling geared towards accelerometer-based classification of road defects. Prior studies in this domain, utilizing machine learning or deep learning for the identification of road defects, have consistently encountered challenges in amassing substantial datasets across diverse environments, primarily due to the complexities associated with data collection and labeling. Our solution, an automated data collection system, streamlines the acquisition and categorization of data, thereby facilitating the generation of comprehensive datasets crucial for deep learning applications.

Secondly, we propose a Convolutional Neural Network (CNN) model specifically designed to leverage this automatically collected dataset. This model is adept at identifying and distinguishing between three common road defects: speed bumps, manholes, and potholes. Through these contributions, our system not only addresses the critical challenges of data collection and labeling in the context of accelerometer-based road defect detection but also presents the potential of deep learning models to enhance the accuracy and efficiency of road defect detection.

### 3.1. An Automatic Data Collection Mechanism for Road Defect Classification

Numerous studies have leveraged deep learning techniques for the classification of road defects through acceleration data. A critical factor in developing an effective deep learning model is the acquisition of accurately labeled data. In this section, we introduce a novel system designed for the automated collection of training datasets tailored for road defect classification. This system significantly enhances the capacity for gathering extensive datasets across varied environments, which is instrumental in improving the deep learning model’s reliability and generalization capabilities.

The proposed system offers a pragmatic and cost-effective solution for data collection, relying on dashcams and smartphones—devices already prevalent in most vehicles. This approach not only streamlines the process of dataset accumulation for training and testing deep learning models but also aligns with the practical constraints of research efficiency and budget. By simplifying the data collection process, our system facilitates a more robust and comprehensive exploration of road defect classification through deep learning, setting a new standard for research in this field.

**Figure 1:**
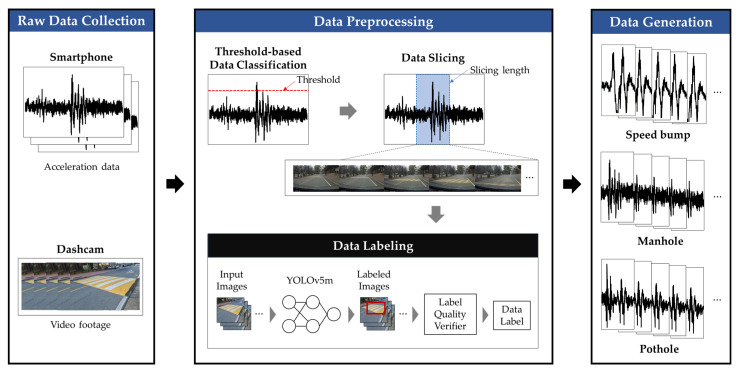
The architecture of automatic data collection mechanism for road defect classification.

Figure 1 depicts the architecture of the proposed automatic data collection mechanism designed for road defect classification. Initially, the system gathers raw data through the accelerometers integrated into smartphones, along with footage from vehicle-mounted dashcams, which serve as the input for the system. Subsequently, in the second phase, both the collected acceleration and video data are segmented and annotated with relevant labels. In the final stage, the labels generated during the data labeling process are amalgamated with the preprocessed acceleration data to formulate a comprehensive dataset suitable for deep learning analysis. This structured approach ensures the efficient collection and preparation of data, facilitating the development of a robust model for road defect classification.

#### 3.1.1. Raw Data Collection

To facilitate the classification of road defects, our methodology employs the accelerometer of smartphones and dashcam video footage as sources of raw data. This approach is notably practical, allowing for widespread application without the necessity for specialized equipment. We have developed an Android application specifically for the purpose of raw data acquisition from smartphones. This application captures data from the three-axis acceleration sensor, along with time stamps, at a sampling rate of 100 Hz. Given the variability in smartphone placement and orientation within the vehicle, a challenge arises in maintaining data consistency.

**Figure 2:**
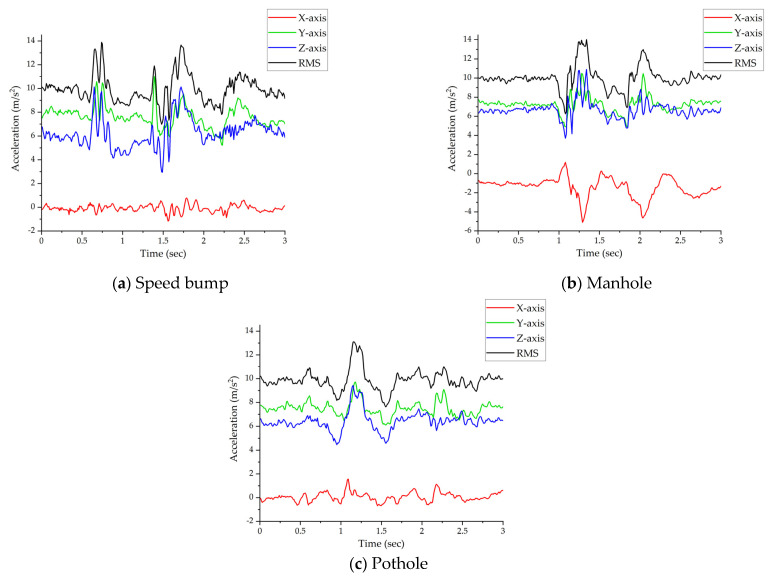
Acceleration X−axis, Y−axis, Z−axis, and RMS values: (**a**) when the vehicle passes the speed bump, (**b**) when the vehicle passes the manhole, and (**c**) when the vehicle passes the pothole.

To address this issue, we compute the root mean square (RMS) value of the acceleration sensor’s x, y, and z axes to standardize the data collection process, as delineated in Equation (1). This methodology ensures that acceleration values are consistent and comparable, irrespective of the smartphone’s position or orientation within the vehicle, as illustrated in Figure 2. The equation for calculating the RMS value of the accelerometer data is as follows:

(1) $${ACC}_{RMS}=\sqrt{({{ACC}_{X})}^{2}+{\left({ACC}_{Y}\right)}^{2}+{\left({ACC}_{Z}\right)}^{2}}$$

Speed bumps, which rise above the road surface, create a distinct signature in the accelerometer data, characterized by an initial increase and subsequent decrease in the accelerometer’s RMS values. This dual-pattern occurrence is due to both the front and rear wheels of the vehicle passing over the bump, as depicted in Figure 2a. Conversely, manholes and potholes, which are depressions in the road surface, generate an inverse pattern in the accelerometer data. This pattern, showcased in Figure 2b,c, is marked by a decrease followed by an increase in the accelerometer readings, corresponding to the vehicle’s wheels dipping into and then emerging from these indentations. Similar to speed bumps, this pattern repeats twice for each vehicle wheel engaging with the defect.

Despite the clear patterns each type of road feature produces, distinguishing between manholes and potholes using accelerometer data alone is challenging due to their similar patterns. Nevertheless, these differences, albeit subtle, can be effectively discerned using deep learning models that have been trained on comprehensive datasets. Such models analyze the nuanced variations in acceleration and deceleration patterns caused by the vehicle’s interaction with these road anomalies, enabling the classification and differentiation of each road feature type based on their unique impact on the vehicle’s accelerometer data.

#### 3.1.2. Data Preprocessing

The data collected from the raw data collection step are preprocessed to generate deep learning analyses. The data preprocessing step first extracts the raw data collected to identify segments indicative of road defects. This is achieved through a threshold-based classification technique, where significant fluctuations in acceleration sensor readings suggest potential road defects. The threshold values used for this determination are established through experimental methods.

If the acceleration value of the raw data exceeds the threshold, both the acceleration values and dashcam video segments are trimmed to lengths that contain road defect information. This trimming, or “data slicing”, leverages the temporal data captured by the acceleration sensors and dashcam footage to ensure precise segmentation. The extent of each data slice is calculated based on the vehicle’s speed and the estimated length of the road defect, aiming to cover the entire duration a vehicle traverses a defect. Utilizing the maximum known speed bump length of 3.6 m [39] and a minimal vehicle speed of 5 km/h (or 0.277 m/s), the slicing length is determined to be approximately 3 s to accommodate the defect passage duration:

(2) $$L_{slice}=\frac{L_{defects}}{S_{min}}=\frac{3.6\;m}{0.277\;m/s}=2.60869\;s≅3\;s\;$$

This procedure ensures a uniform dataset, with each slice containing 300 data points, given the 100 Hz sampling rate of the sensor. For video data, only the initial second of footage, prior to encountering a road defect, is utilized for labeling, resulting in 30 video frames per event for analysis.

Addressing the model training aspect, while unsupervised learning offers the benefit of identifying patterns without labeled data, it often lacks the interpretability and accuracy of supervised learning models, which rely on high-quality labeled data. The challenge lies in the labor-intensive and costly process of acquiring accurately labeled data. To mitigate this, our approach incorporates automatic labeling of sensor data using dashcam footage, significantly reducing the cost and effort compared to traditional manual labeling techniques.

For precise defect labeling, we employ the YOLOv5m [40] model, which specializes in image classification, to analyze dashcam images synchronized with sensor data. The preliminary step in developing a robust road defect classification model with YOLOv5m involves the acquisition of accurately labeled images. While obtaining these labeled images is crucial, it is notably less challenging and resource-intensive compared to the process of collecting and labeling sensor data. We have successfully developed a YOLOv5m model that demonstrates over 90% accuracy in identifying specific road conditions such as speed bumps, manholes, and potholes. To mitigate this risk, we employ a stringent data validation approach. During a second of video capture, 30 dashcam images are extracted and individually assessed using the YOLOv5m model. Only when at least 90% of these images yield consistent classification results is the data considered high-quality and retained for further processing. This method ensures that only data of the highest integrity are used for training our model, effectively enhancing the model’s reliability and performance by excluding low-quality or ambiguous data points. Through this rigorous label quality verifier, we are able to generate a high-quality dataset for the training of our deep learning model.

#### 3.1.3. Data Generation

The final step is to combine the 3-s sliced acceleration data with labels derived from the data preprocessing step. This composite dataset forms the foundational input for constructing deep learning models aimed at road defect detection. Within the scope of this study, we have focused on generating labeled data for three specific road defects: speed bumps, manholes, and potholes. However, the versatility and efficiency of our automated data collection system facilitate the easy and effective expansion of this dataset to include a wider array of road defect types. This capability underscores the system’s potential to significantly contribute to the development of more comprehensive and accurate road defect detection models, leveraging deep learning to improve the safety and maintenance of transportation infrastructure.

### 3.2. A Road Defect Detection with 1D-CNN

Recurrent Neural Networks (RNNs) are especially adept at handling temporal or sequential data, making them ideal for tasks involving continuous time series or textual data. These models possess the unique capability to remember and integrate past information with incoming data, enabling them to perform effectively in scenarios where the sequence and context of data points are critical. RNNs are particularly effective in both classification and prediction tasks. Although these tasks might appear distinct, prediction plays a crucial role in enabling RNNs to develop a nuanced internal representation of the input sequence’s essential characteristics. This capability significantly enhances the RNN’s training efficiency, accuracy, and generalization to new data. As a result of this sophisticated processing ability, RNNs demonstrate superior classification performance, providing users with a reliable means to assess the outcome of classification tasks [41].

Long Short-Term Memory (LSTM) networks, a subtype of Recurrent Neural Networks (RNNs), are designed to solve the problem of long-term dependencies in sequence data. Their unique architecture enables the retention of information over long periods, making them ideal for complex time series analysis. A novel approach combines LSTM with Fully Convolutional Networks (FCN) for time series sequence classification, resulting in the LSTM-FCN model. This model outperforms standard FCN models in accuracy while maintaining a small increase in size and minimal data preprocessing requirements. Further enhancing this model, the Attention LSTM Fully Convolutional Network (ALSTM-FCN) incorporates attention mechanisms to improve classification performance and allows for the visualization of LSTM decision processes, making it a sophisticated tool for time series analysis with enhanced interpretability [42].

The One-Dimensional Convolutional Neural Network (1D-CNN) proves to be a powerful tool for identifying patterns within time series data, making it particularly useful in fields like signal processing and audio analysis. In the context of time series classification, the selection of an appropriate kernel size is pivotal, as it significantly influences the model’s performance. To address the challenge of determining the optimal kernel size, this paper introduces a novel architecture, the Omni-Scale 1D-CNN (OS-CNN). This architecture is designed to dynamically identify and adapt the kernel size during the training process, thereby enhancing the model’s ability to extract relevant features from time series data across different scales. The OS-CNN represents a significant advancement in the utilization of 1D-CNNs for time series analysis, offering a flexible and efficient approach to capturing the complexities of temporal data [43].

**Figure 3:**
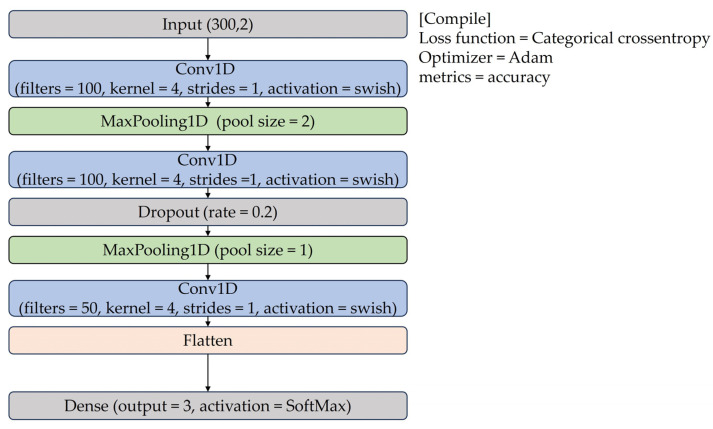
Structure of RDD-CNN model.

In this paper, we propose road defect detection with a 1D-CNN (RDD-CNN) model. As Figure 3 presents the one-dimensional convolutional layer extracts spatial features from time-series data, while 1D Max Pooling reduces data dimensions, and Dropout prevents overfitting. The flattened layer transforms multi-dimensional feature maps into a one-dimensional vector, and the dense layer performs final classification or prediction. The Swish function is used in the one-dimensional convolutional layer, with filter sizes of 100 and 50, a kernel size of 4, and strides of 1. The final dense layer employs a SoftMax activation function, a categorical cross-entropy loss function, and an Adam optimizer. The training dataset for the RDD-CNN model uses generated data from the Automatic Data Collection System.

The continuous acceleration sensor data collected while driving the vehicle is tested using our proposed RDD-CNN model. To classify three distinct road defects (i.e., speed bumps, manholes, and potholes) from the continuous data, we propose a sliding window algorithm as in Algorithm 1. This algorithm is designed to capture and analyze sequential patterns in the data, recognizing the inherent connectivity between each data point and its predecessors. The sliding window, set to a duration of 3 s, advances in increments of 0.1 s across the dataset. Within each window, the acceleration data are processed by the RDD-CNN to determine if it corresponds to one of the three targeted road defects. Upon successful classification, the algorithm adjusts such that the ending point of the current window becomes the starting point for the subsequent window. This approach allows for the continuous and dynamic analysis of sensor data, enabling the RDD-CNN to effectively distinguish between different road defects based on the characteristics of the recorded acceleration patterns.
**Algorithm 1.** Sliding window algorithm.**Sliding Window Algorithm**procedure SLIDINGWINDOW (Accelerometer RMS Data, window size, overlap)  start ← 0  end ← window size **  while** end ≤ length(Accelerometer RMS Data) **do**   current window ← extract window(Accelerometer RMS Data, start, end)   detection result ← apply detection algorithm(current window)   **if** detection result **then**       process detection(current window)       start ← end       end ← start + window size   **else**   start ← start + window size − overlap   end ← end + window size − overlap   **end if**  **end while** **end procedure**

To operate the RDD-CNN model on smartphones, the model should be lightweight and suitable for the computational constraints of mobile devices. To this end, among various strategies for model optimization, we employ bit quantization as a method to reduce the model’s computational footprint. Specifically, we convert 32-bit floating-point numbers to 16-bit integers, a process known as quantization. Quantization significantly enhances the computational efficiency of CNN by accelerating processing speed and diminishing memory requirements [44]. This method is applied to the weight matrices of both the convolution layer filters and the fully connected layers, aiming to retain the model’s accuracy by minimizing the response error associated with the reduction in numerical precision. The implementation of this quantization technique enables the operation of the RDD-CNN model on smartphones, thereby facilitating real-time road defect detection directly from a user’s mobile device.

## 4. Experimental Results

### 4.1. Experiment Setup

**Figure 4:**
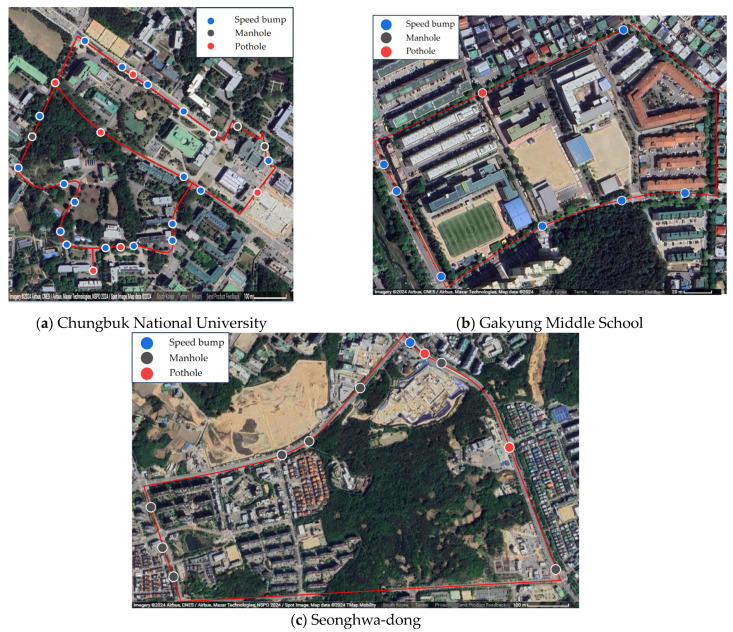
Dataset collection location: (**a**) training dataset collection location; (**b**,**c**) test dataset collection location.

In this study, three Android smartphones—the Samsung Galaxy Note8, Xiaomi Redmi Note 10 Pro, and LG Q7—were used to collect raw accelerometer data. The accelerometer data are saved in an Excel file comprising multiple columns, including timestamp, accelerometer X-axis, accelerometer Y-axis, accelerometer Z-axis, and calculated RMS of the accelerometer. Samsung Galaxy Note 8 CPU is Octa-core (4 × 2.3 GHz Mongoose M2 and 4 × 1.7 GHz Cortex-A53)—EMEA, GPU uses Adreno 504. The CPU of the Xiaomi Redmi Note 10 Pro uses Octa-core (2 × 2.3 GHz Kryo 470 Gold and 6 × 1.8 GHz Kryo 470 Silver), and the GPU uses Adreno 618. The LG Q7 CPU uses an ARM Cortex-A53 Octa-Core 1.8 GHz CPU, and the GPU uses an Adreno 506 400 MHz GPU. To collect raw video data, an INAVI QHD5000 dashcam equipped with a 5.14 M Pixels, 1/2.8” (CMOS) sensor on both front and rear cameras is utilized, enabling the storage of video at a resolution of 2560 × 1440 and a frame rate of 30 frames per second. The video files are saved as mp4 files, incorporating the recording timestamp. We collect these raw data using two vehicles equipped with these devices: a YF Sonata and a Kia All New Sportage. The YF Sonata is a sedan, and the Kia All-New Sportage is an SUV. The criteria for choosing a data collection site were based on a high number of road defects and the safety of experiments. In Cheongju, South Korea, near Chungbuk National University, we conducted a total of 20 h of driving covering 300 km to collect raw data for training deep learning models, as illustrated in Figure 4a. Moreover, for model testing purposes, we gathered additional raw data through 8 h of driving, covering 120 km in the vicinity of Gakyung Middle School and Seonghwa-dong, Cheongju, South Korea, as depicted in Figure 4b,c.

In the data preprocessing step, we utilized a Linux environment and Python version 3.8.10. The threshold is set to 12 m/s^2^ for the threshold-based data classification. The slicing length is set to 3 s for the data slicing. To develop the YOLOv5m model used for data labeling, we utilized an open data source [45] and directly collected images, including 3000 for speed bumps, 3500 for manholes, and 4000 for potholes. The size of the YOLOv5m model is 882 MB. Through the data preprocessing step, we achieved 576 speed bumps, 290 manholes, and 271 pothole datasets with 100% accurate labels. However, 13 speed bumps, 68 manholes, and 69 pothole datasets were discarded as they could not pass the label quality verifier.

The RDD-CNN model was constructed using TensorFlow 2.9.1. For model training, a total of 696 datasets were utilized, while 300 datasets were employed for testing purposes. In the input layer of the model, data consisting of 300 rows and a single column representing the RMS value of the accelerometer sensor data, forming a (300,1) dimension, was processed through a 1D convolution layer. In the output layer, the model was trained using the SoftMax activation function, the categorical cross-entropy loss function, and the Adam optimizer. The training process spanned over 30 epochs, with a batch size set to 15. Finally, the TFLite Converter from the Android TensorFlow Lite library was employed to generate a lightweight version of the RDD-CNN model. The optimized model is designed for execution on smartphones, including the Samsung Galaxy Note8, Xiaomi Redmi Note 10 Pro, and LG Q7, ensuring broad accessibility and practical deployment in mobile applications.

### 4.2. Evaluation Results of the Automatic Data Collection System

The raw data undergo a process of threshold-based data classification and data slicing to isolate data segments containing road defects. In the threshold-based data classification, the acceleration threshold was set to 11 m/s^2^ through iterative experiments to maximize the detection of road defects. Table 2 shows the discarded data ratio by the threshold-based data classification. Despite the presence of actual road defects, the results of undetected road defects by the threshold-based data classification are as follows: out of 604 speed bumps, 15 were not detected; out of 376 manholes, 18 were not detected; and out of 361 potholes, 21 were not detected. The 15 undetected speed bumps were all cases where the bump was simply painted on the road without any physical undulation. The undetected 18 manholes and 21 potholes were cases where the defects were not severe enough to cause significant vehicle vibrations. This highlights the limitation of threshold-based data classification, which relies on accelerometers and cannot classify defects with relatively mild undulations that do not cause noticeable changes in road surface level.

**Figure 5:**
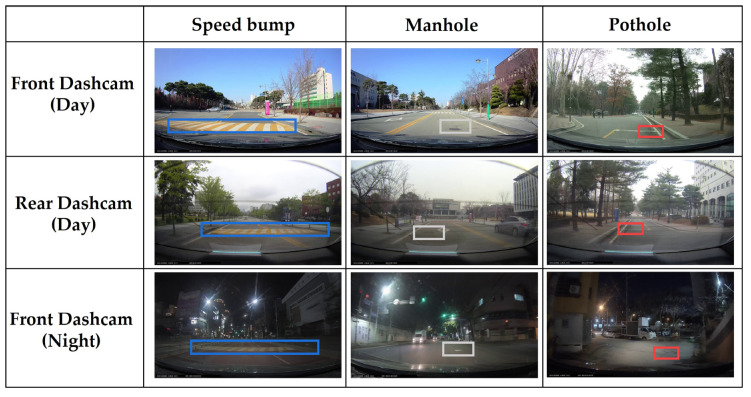
YOLOv5m-based road defect classification samples.

Data exceeding the threshold value are subject to a data-slicing phase. Each sliced data segment comprises 300 acceleration data and 30 video images. To automatically generate labels for each sliced data segment, the 30 video images are analyzed using the YOLOv5m model. In an initial evaluation, the YOLOv5m model was applied to a comprehensive dataset consisting of 522 images, each collected by the front and rear dashcams during the day, and 356 images collected by the front dashcam during the night, for a total of 1400 images. The experiment was conducted during the day when there was no snow or rain, the sun was shining, or there were some clouds. The reason is that it is dangerous to collect data when it is snowing or raining, so it was conducted during the day when the weather was good. Table 3 shows significant performance gaps between day and night conditions, as well as between front and rear dashcams. The rear dashcam demonstrated significantly low performance in detecting road defects at night, warranting its exclusion from the table, while the front dashcam at night showed considerably low quality, as seen in Figure 5. The blue square represents the speed bump, the gray square represents the manhole, and the red square represents the pothole. These findings indicate that vision-based road defect detection techniques are highly sensitive to environmental conditions, rendering them unsuitable for reliable detection. The accuracy of road defect classification from data collected by the front dashcam during the day is, on average, 24.27% higher than that of the rear dashcam. This decline is primarily due to the lower image quality of the rear dashcam and the obstruction caused by the defroster lines on the vehicle’s rear window. Therefore, the proposed automatic data collection mechanism limits the collection of raw data to daytime conditions using video from front dashcams. This approach is adopted to enhance the reliability and quality of the generation data.

In the data labeling phase, testing a single video image with the YOLOv5m model yields an average accuracy of 95.17%. Although this accuracy rate is relatively high, it is still considered insufficient for use as a label in training data. To overcome this limitation, we implemented the label quality verifier technique, which assigns a single label based on aggregated results from multiple video image analyses. This method ensures that a label is adopted only if the consensus among the results surpasses a classification threshold. Any dataset not meeting this threshold is subsequently discarded. Table 4 presents the experimental results on the proportion of data discarded and the accuracy of labels according to classification thresholds. As the classification threshold increases, the amount of discarded data also increases, but this leads to higher label accuracy, thereby generating more reliable data. To maximize the collection of accurate data, we utilize data defined with a 90% classification threshold for training the deep learning model.

**Figure 6:**
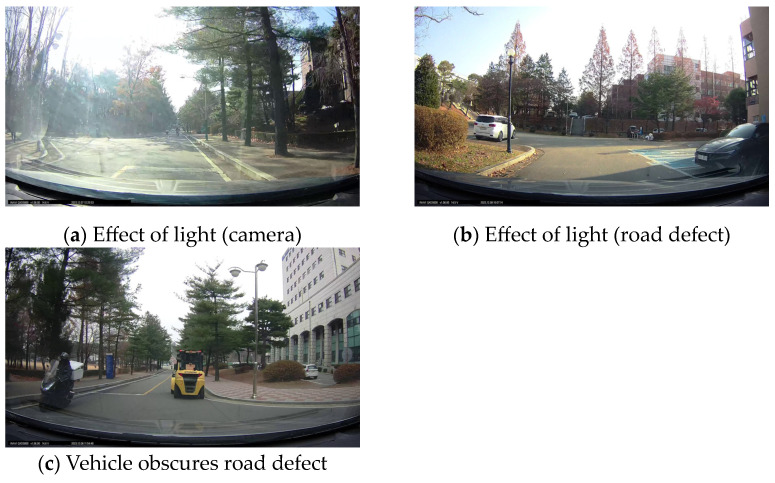
Environmental factors obstructing video image classification: (**a**) when excessive light enters the camera; (**b**) when excessive light enters a road defect; and (**c**) when another vehicle obscures a road defect.

Upon analyzing the images of discarded data, it was discovered that a significant number of instances failed to be classified by the YOLOv5m model for environmental reasons depicted in Figure 6. In the case of speed bumps, their extensive coverage area resulted in fewer instances being obscured by light effects or obstacles. Conversely, manholes and potholes, characterized by smaller areas of defect, were more frequently affected by light and obstacles, leading to an increased rate of data exclusion. Beyond environmental factors, the YOLOv5m model exhibited lower classification accuracy for manholes and potholes compared to speed bumps, contributing to a higher proportion of data being discarded due to the label quality verifier’s criteria. Due to these reasons, as indicated in Table 5, with a 90% classification threshold set by the label quality verifier, 13 out of 589 data points for speed bumps, 68 out of 358 data points for manholes, and 69 out of 340 data points for potholes were discarded.

### 4.3. Evaluation Results of the RDD-CNN

In this subsection, we evaluate the performance of road defect detection models. The performance of the RDD-CNN model trained on manually generated data by humans versus data produced by automatic data collection is evaluated. As shown in Table 6, the performance evaluation revealed a negligible error rate of 1%. The model trained on automatically generated data showed slightly lower accuracy due to a reduced number of data points, resulting from the system discarding certain data. The manually collected dataset comprised a total of 1287 data, while the automatically generated dataset contained 1137 data. As the volume of collected data increases, this disparity is expected to decrease.

Table 7 illustrates the impact of a smartphone’s placement within a vehicle on its accuracy in detecting road defects. The results indicate that there was no discernible difference in accuracy when the smartphone was positioned either on the dashboard or the passenger seat, likely due to the stability provided in these locations. However, the accuracy diminished when the smartphone was placed in less stable locations, such as the cup holder, the door pocket, or the clothes pocket. In these positions, the smartphone experienced more movement, especially when the vehicle traversed road imperfections, leading to increased errors in data collection and, consequently, a reduction in accuracy. This variation in data accuracy based on the smartphone’s location within the vehicle underscores the importance of a stable mounting position for optimal data collection, particularly when utilizing smartphones for detecting road defects. Future research is needed to ensure consistent accuracy regardless of smartphone placement.

**Figure 7:**
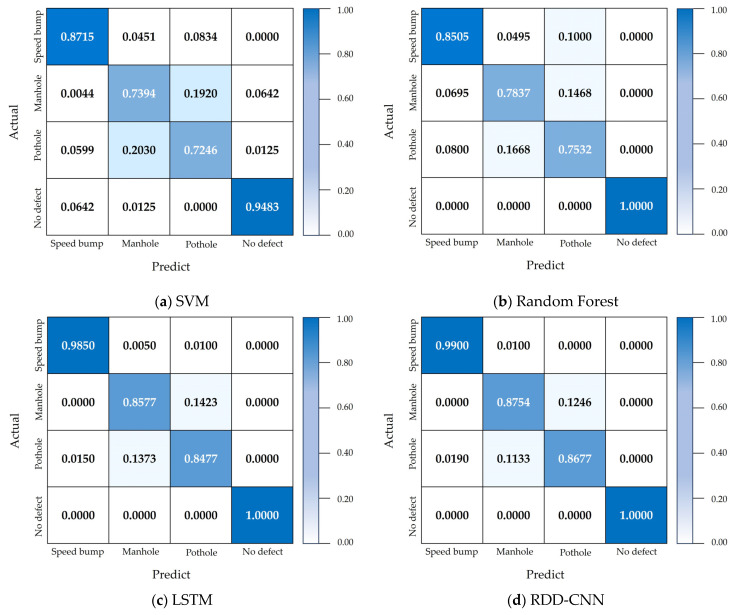
Accuracy confusion matrix for each model: (**a**) SVM confusion matrix, (**b**) Random Forest confusion matrix, (**c**) LSTM confusion matrix, and (**d**) RDD-CNN confusion matrix.

Figure 7 presents a confusion matrix that provides a detailed view of the performance of the SVM model, Random Forest model, LSTM model, and RDD-CNN model when utilizing automatically generated. The results indicate that RDD-CNN is exceptionally adept at distinguishing between speed bumps and no-defect scenarios, achieving an accuracy of 99%. This precise discrimination is largely due to the significant variance in vehicle motion induced by speed bumps as opposed to other defect types. On the other hand, potholes and manholes, which are generally characterized by their recessed shapes, offer a more subtle challenge for distinction, hinging on the depth and shape of the depression. Manholes usually feature a relatively even surface, whereas potholes are characterized by more pronounced irregularities and indentations, as shown in Figure 2. The similarity in the physical characteristics of potholes and manholes leads to a comparatively lower performance in the classification of these two types of road defects.

The models of road defect detection under comparison include SVM, Random Forest [46], Long Short-Term Memory (LSTM), and our proposed RDD-CNN, each utilized for estimating road defect conditions. SVM is a supervised learning algorithm widely employed for both classification and regression challenges. It aims to identify a hyperplane that maximizes the margin between different classes, with the margin defined as the distance between the nearest data points of any class (known as support vectors) and the hyperplane itself. The training process for SVM involves solving a quadratic optimization problem subject to boundary constraints and a linear equation constraint. However, traditional optimization methods for such problems are often deemed impractical due to the extensive memory and time they require [47].

Random Forest, on the other hand, is an ensemble learning technique applicable to both classification and regression tasks. It operates by constructing multiple decision trees during the training process and merging their outcomes to produce more accurate and robust predictions. This method effectively mitigates the risk of overfitting and is suitable for diverse datasets. The strategy of integrating predictions from several decision trees, particularly when the number of variables significantly surpasses the number of observations, has been acknowledged for its efficacy. Random Forest is also scalable, making it appropriate for large-scale problem-solving scenarios [48].

These established methods, along with LSTM—a neural network designed for processing sequences and temporal patterns—provide a comprehensive backdrop against which the performance of the proposed RDD-CNN model is assessed. The comparison aims to highlight the strengths and potential of RDD-CNN in accurately detecting road defects using data derived from the Automatic Data Collection System. Table 8 presents the specific parameter values utilized for each model in the detection of road defects.

**Figure 8:**
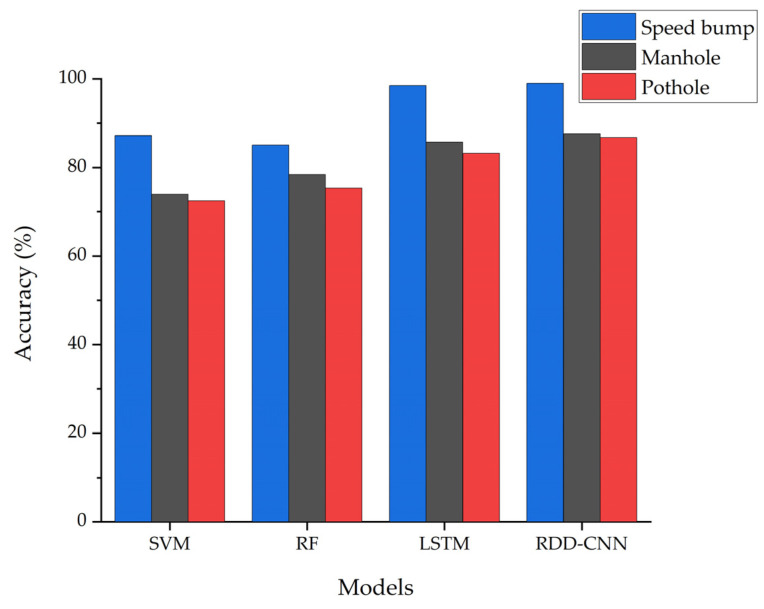
Accuracy of various testing models.

Figure 8 illustrates the accuracy of artificial intelligence models utilized in research for detecting road defects. Deep learning models such as LSTM and CNN demonstrated relatively higher accuracy compared to traditional machine learning models like SVM and RF. This superiority of deep learning models can be attributed to their ability to learn high-level abstract features from data, enabling them to understand complex patterns and relationships. In contrast, traditional machine learning methods rely on predefined features, mostly designed manually, which may not capture the complexity present in large datasets as effectively as the features learned automatically by deep learning models. Most existing studies on road defect detection classify accelerometer data to identify road defects. While LSTM is adept at modeling temporal sequences and dependencies in time-series data, CNNs can be more efficient when the data length is short or the patterns are relatively simple. Therefore, in classifying defects through accelerometer data input, CNNs offer advantages, which is why RDD-CNN shows higher accuracy.

**Figure 9:**
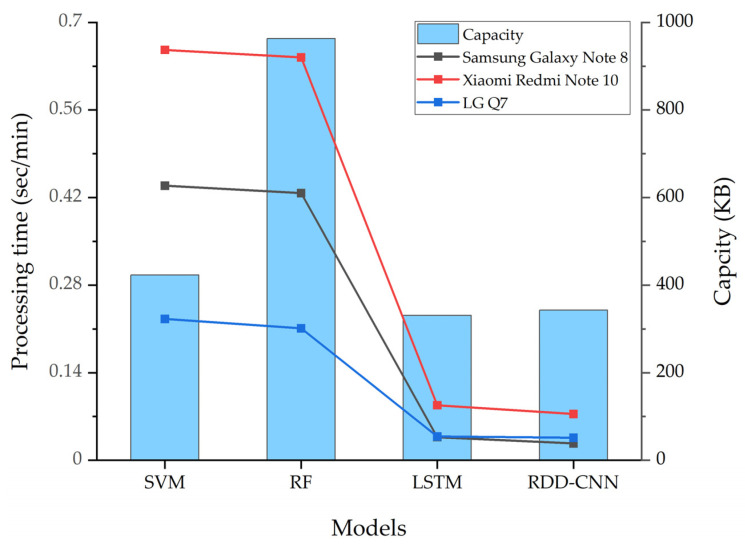
Comparison of lightweight size and processing time for each model.

This paper aims to detect road defects using smartphones, making it crucial to maintain high detection accuracy while reducing the load on smartphones. To achieve this, we performed quantization to lighten the model, reducing its size by an average of 59.11%. Figure 9 presents the average time required by each model to evaluate 1-min test data using the sliding window algorithm and the size of each model. The proposed sliding window algorithm reduces the number of tests when road defects are present in the test data and increases the number of tests when there are no road defects. Through experiments with various data inputs, it was found that each model performed an average of 533.75 tests per minute. SVM can implement more accurate models with smaller tol values but requires significantly longer processing time compared to deep learning approaches. Random Forest also shows improved accuracy with an increased number of decision trees, but at the cost of longer computation times. LSTM and RDD-CNN exhibited relatively short processing times compared to traditional machine learning techniques. Quantization did not lead to differences in accuracy but allowed for a reduction in processing time, enhancing the feasibility of using these models on smartphones.

## 5. Conclusions

This paper introduces a system that leverages smartphones to identify road defects, featuring an innovative automatic data collection mechanism and the RDD-CNN model, proficient in real-time road defect detection. This mechanism greatly facilitates the data acquisition process, enabling the automatic collection of data and label information solely from road driving videos and sensor data, thereby enhancing convenience in data collection. The experimental results, conducted with three smartphones in two vehicles, revealed that the automatic data collection method missed only 15.21% of the data while achieving 100% label accuracy.

The testing of the RDD-CNN model, our proposed road defect classification model, with automatically collected data exhibited a negligible error margin, only 0.4% lower than that with manually collected data. The evaluation showcases the feasibility and effectiveness of employing automatically gathered data for training various AI models. Notably, the RDD-CNN model demonstrated superior classification accuracy, exceeding 86.77% compared to other models, and is optimized for smartphone use, maintaining a low processing time of approximately 0.1 s per minute of driving, confirming its real-time operational capability.

The scope of this research is currently limited to classifying road defects in real-time on local smartphones. For future developments, we aim to utilize the technology to update a cloud server with real-time locations of road defects, creating a live defect map. By using crowdsourced data from the cloud server, we plan to achieve more precise road defect classifications, thus improving the overall accuracy and reliability of road defect detection and mapping, with considerations for the server’s storage and bandwidth capacities.

## Figures and Tables

**Table 1:** The summary of road defect detection technologies.

Method	Strengths	Weaknesses
Vision-based method	The number and shape of road defects can be known.	The camera affects light and shadow.
3D reconstruction method	The shape and depth of road defects can be accurately determined.	The price of equipment is high.
Vibration-based method	It is the cheapest when compared to a vision-based method and a 3D reconstruction method.	It is difficult to determine the shape or depth of road defects.

**Table 2:** The ratio of discarded data by the threshold-based data classification.

	Speed Bump	Manhole	Pothole
Ratio of discarded data	2.483%	4.787%	5.817%

**Table 3:** Performance comparison of YOLOv5m-based road defect classification in various environments.

Experiment Environment	Speed Bump	Manhole	Pothole
Front Dashcam (Day)	98.4%	94.8%	92.3%
Rear Dashcam (Day)	82.6%	68.3%	61.8%
Front Dashcam (Night)	78.3%	52.5%	43.8%

**Table 4:** The ratio of discarded data and the label accuracy according to classification thresholds by the label quality verifier.

Classification Thresholds	Ratio of Discarded Data	Label Accuracy
100%	14.60%	100%
90%	11.65%	100%
80%	9.86%	98%
70%	4.21%	96%
60%	3.03%	95%

**Table 5:** The ratio of discarded data by the label quality verifier.

	Speed Bump	Manhole	Pothole
Ratio of discarded data	2.207%	18.99%	20.29%

**Table 6:** Performance of RDD-CNN model according to types of training datasets.

Types of Training Datasets	Speed Bump	Manhole	Pothole
Manually generated data	99.00%	88.46%	87.29%
Automatically generated data	99.00%	87.54%	86.77%

**Table 7:** Average accuracy according to the placement of the smartphone in the vehicle.

	Dashboard	Passenger Seat	Cup Holder	Door Pocket	Clothes Pocket
Accuracy	91.10%	90.76%	84.61%	83.07%	82.85%

**Table 8:** Hyperparameters of testing models.

Models	Hyperparameters
SVM [46]	Kernel = linear, C = 1.0, shrinking = true, tol = 0.001, random state = 0
Random Forest [46]	N estimators = 10, max depth = none, min samples split = 1, random state = 0
LSTM [49]	Input shape = (300,1), learning rate = 0.001, activation = SoftMax Optimizer = Adam, loss function = categorical cross entropy, dropout = 0.5
RDD-CNN	Input shape = (300,1), activation = Swish, kernel size = 4, loss function = categorical cross entropy, optimizer = Adam

## Data Availability

The road defect detection dataset is available at https://github.com/GyuLimkim/Road_Defect_Dataset/tree/main (accessed on 21 March 2024).
